# Two Cases of Popliteal Aneurism, Cured by Compression of the Trunk of the Artery at the Cardiac Side of the Tumour, with Some Remarks on That Method of Treating the Disease

**Published:** 1846-09

**Authors:** William Henry Porter

**Affiliations:** Professor of the Theory and Practice of Surgery in the Royal College of Surgeons in Ireland; Surgeon to the Meath Hospital and County of Dublin Infirmary; and consulting Surgeon to the City of Dublin Hospital


*Two Cases of Popliteal Aneurism, cured by Compression of the Trunk of the Artery at the cardiac Side of the Tumour, with some Remarks on that Method of treating the Disease.* By WILLIAM HENRY PORTER, M. D., Professor of the Theory and Practice of Surgery in the Royal College of Surgeons in Ireland; Surgeon to the Meath Hospital and County of Dublin Infirmary; and consulting Surgeon to the City of Dublin Hospital.

William Devereux, a tailor, æt. 29, was admitted into the Meath Hospital December 3, 1844, when he stated that he had generally enjoyed excellent health, until, about two months previous to his application for relief, when he observed an unusual pulsation or throbbing in the right ham, which, however, gave him no uneasiness, except that he imagined that limb to be occasionally stiff in walking. He thus continued for six weeks, at the end of which a tumour appeared at the spot where he first observed the throbbing, which gradually increased until it attained the size of a small orange, though still unattended with pain; but the size of the swelling and the stiffness produced by it, interfering with the power of extending the limb, he applied sixteen leeches which he imagined had some effect in reducing its dimensions. During the last two days it increased so rapidly as to create alarm, and induce him to apply for assistance.

On examination, a tumour was observed pulsating strongly in the right popliteal space, and occupying its entire extent, firm and incompressible externally, but softer internally, where pressure had the effect of diminishing its volume considerably. The expansion during its diastole was equally perceptible in every direction, and a loud *bruit de soufflet* was heard over every part of it. Pressure on the artery at the groin reduced its size by nearly one-third. The man's general state of health appeared to be very good, and he made no complaint except of pain occasionally shooting down to the external ancle, particularly at night.

Dec. 6. At half past ten o'clock this morning I applied pressure on the trunk of the femoral artery by means of two clamps: one applied about two inches below Poupart's ligament, the other at a spot nearly corresponding to the place where the vessel enters the tendon of the triceps muscle, with the intention that one might be screwed down when it became necessary that the other should be relaxed, and thus a constant and uninterrupted compression be maintained. The superior clamp was then screwed down until the impulse of the circulation was communicated to the instrument, which vibrated with each stroke of the vessel. Under this pressure the pulsation of the tumour was scarcely, if at all, diminished, and he bore it without apparent inconvenience for a quarter of an hour, when it was relaxed—the inferior clamp having been previously screwed so as to produce a corresponding effect; but it was found that he could not endure this latter either so well, or for such length of time as the former, the reason of which appeared to be that a comparatively much greater force was necessary to produce a given effect on the circulation when used where the vessel was deeply covered, and consequently the pressure on the soft parts there was stronger and less endurable. In the evening the limb was slightly swollen and discoloured, and he complained of pain in the calf of the leg, which latter symptom was relieved by wrapping the part in flannel.

Dec. 7. Passed a sleepless night; the limb more swollen but less painful.

Dec. 8. Had some little sleep from the effects of an opiate. The pain was now trifling, but the tumefaction increased, and there was some œdema. On this day he was able to endure the pressure of the superior clamp for half an hour, and of the inferior for twenty minutes; neither instrument was ever screwed so tightly as completely to obstruct the circulation, or stop the pulsation in the tumour.

Dec. 10. On this morning I found both the clamps loose and unscrewed: this had been done by the patient himself, who was feverish, irritable, and discontented, complaining of headach, and having a hot skin and accelerated pulse. He was ordered some aperient medicine, which afforded great relief.

Dec. 12. I found it nearly impossible to affect the circulation of the limb by the inferior clamp: it could not be brought to bear directly on the vessel, and consequently the degree of pressure used was too forcible without being very effective. Mr. Millikin, the ingenious instrument maker, of Parliament street, contrived a clamp in which this objection was obviated: it answered the purpose admirably.

Dec. 13. The œdema of the limb had nearly disappeared, and there was no pain; the *bruit de soufflet* scarcely perceptible in the tumour, and the force of the pulsation diminished.

Dec. 15. The case seemed to be progressing most favourably when, unfortunately, the new instrument broke, and pressure of any kind was discontinued for several hours. The symptoms all returned as in the commencement.

Dec. 16. A new instrument having been constructed, the pressure was resumed, the symptoms being as strongly marked as at any previous period, but the general condition of the patient much more favourable; he could now endure the operation of the superior clamp uninterruptedly for an hour and a half, that of the inferior one for an hour, and neither occasioned œdema, discoloration, or pain.

Dec. 20. The *bruit de soufflet* was no longer audible, and the tumour seemed to be firmer and smaller. The patient appeared perfectly to understand the object of the treatment, which had been frequently explained to the pupils at his bed-side, and managed the screws of the instrument himself with considerable judgment: the necessary attendance on him was, of course, diminished, and the case afterwards gave but little trouble.

Dec. 30. On the evening of this day he suddenly exclaimed that the pulsation of the ham had ceased, and that he was cured. On examination such was found to be the case.

Jan. 6. The tumour was hard, diminished in size, and totally devoid of pulsation. He was up on this day, weak and somewhat emaciated from confinement, but in other respects perfectly well. He has since been frequently seen by me, and by several of the pupils of the hospital; and up to the last day on which he came under observation (July 19, 1845,) he remained without the slightest apparent tendency to a recurrence of the disease. The limb that had been engaged exhibited no difference from the other, except that, on minute examination, a small, firm, kernel-like tumour could be felt deep in the popliteal space.

The next case is that of a medical gentleman, who was under my care last summer, and is particularly interesting from the circumstance of the treatment having been discontinued long before recovery took place—so long, that many may be disposed to doubt the validy of pressure in this case at all. For this reason I requested my patient to furnish me with his own notices of the case, and I give them in his own words: they are perhaps, less methodically arranged, and less circumstantial, than might be desired, but their authenticity cannot be questioned; and it may be added, that however sceptical others may prove as to the influence of the treatment employed, Dr. Murray never for a moment attributed his recovery to any other cause. During the progress of the case it was seen at different times by Doctors Cusack, Hutton, M. Collis, Smyly, Rynd, and I believe, several others.

"Charles James Murray, M. D., while travelling in Italy, about nine months ago, received a strain from having made too long a step when alighting from a carriage. This did not produce much uneasiness or inconvenience at the time; but having, on two or three subsequent occasions, overstretched the leg, he began to feel at intervals, sometimes of two or three weeks, sensations as of cramp in the upper part of the thigh, and stiffness in the knee. About four months afterwards (and, in the mean time, he had been in the daily habit of taking a great deal of exercise on foot,) the feeliug of stiffness became permanent, and was greatly aggravated by flexion of the knee, but relieved by walking or moving about.

"On directing his attention more particularly to the part, he discovered a decided fulness in the popliteal space, with a violent pulsation; and he also observed that the veins of the leg were evidently distended: still, though convinced that an aneurism was forming, he continued to attend to the duties of a dispensary for two months longer, until, perceiving a decided increase in the symptoms, he consulted Mr. Porter (July 28, 1845.)

"*Symptoms.*—The tumour soft, compressible, and circumscribed, occupying a length of about two inches of the vessel.

"Pulsation well marked and decided, and a loud *bruit de soufflet,* attended with a peculiarly rough sound, accompanied each impulse of the artery. Pressure on the vessel at the groin caused the pulsation to cease, and the sac to collapse and appear empty. There had been no increase of the swelling, but the stiffness in the ham had become much more troublesome.

"On the 2d of August pressure was applied on the artery, under Mr. Porter's direction, in a moderate degree, and alternated from one point to another, according to the patient's feelings, but rarely to such a degree as to stop the pulsation in the ham. The action of the heart was very strong and excitable. The patient had cool drinks and a mild nutritious diet. The bowels were kept regularly open.

"The patient's chief complaint during the maintenance of the pressure was of a dreadful burning sensation along the course of the tibia, and this became so severe, that at the end of twenty days he refused to bear it any longer, and moved to the country to recruit his strength There was then a decided hardness in the seat of the disease, but the pulsation appeared to be nearly as great as before.

"On the 27th of September, four weeks from the removal of the pressure (and during a fortnight of this time he had used a good deal of exercise) he felt on getting out of bed an unusual degree of stiffness in the knee, and, on examining, found an increased hardness in the part, and no trace of pulsation. On the previous day he had walked more than usual, and had gone to bed very much fatigued.

"It is only necessary to add that the cure, however accomplished, is a complete one, no symptom of the disease having ever reappeared."

The treatment of aneurism by "compression" has been already so extensively commented on, and so satisfactorily explained, by the several surgeons of Dublin, who have practised it with success, that the mere publication of the preceding cases, and the evidence they afford, might be deemed sufficient, without entering farther on the subject; but having devoted considerable attention to the pathology of this formidable disease, and being frequently consulted by my professional brethren and friends, I find that this, one of the greatest improvements of modern surgery, is really not understood, and, therefore, cannot be duly appreciated. For instance, I have evidence in my possession to shew that by some, compression of the tumour itself has been strangely enough confounded with that applied on the trunk of the artery; by others it has been tried in cases to which it was inapplicable, and in which it could not possibly succeed; and by others it has been improperly and wrongfully employed altogether; so that, although extensively known for some years, and frequently tried, the practice, with the exception of two or three cases, seems only to have been successful in Dublin. Again, I have been asked is not this treatment by compression tedious, irksome, wearisome, and painful; have not patients abandoned it, and allowed the disease to take its course, rather than submit to such protracted suffering; and what possible advantage can it offer over an operation, easy of performance, decisive in its effects, and generally fortunate in its results? These are questions which require full and explicit answers. We know that implicit reliance may not always be placed on the announcements of discoveries in medical science, however confidently put forward, for many such, when exposed to the rigid test of practical investigation, have proved most miserable failures; and we can easily imagine, that a surgeon possessed of great dexterity, and favoured by fortune to an extent not often experienced, should demand the most undeniable evidence, before he could be induced to exchange the operation that in his hands had led to such happy results, for any other mode of treatment whatever. It is, then, with the object of meeting some of those objections, that I offer the following remarks. Calm and careful inquiry seldom fails to elicit truth, and let it be but fairly established, and public opinion will do the rest; it will regulate our conduct in this, as it does in most other respects, but it is a power so stupendous, that a scientific man will seek rather to guide and direct, than merely be compelled to follow it, and will much prefer having his understanding convinced in the first instance, to yielding with a bad grace when opposition shall have become unavailing.

Already has this subject of the treatment of aneurism by compression been examined and explained to an extent that leaves little room for further observation;* already it has been satisfactorily proved to be equally applicable with the ligature to many of the most unpromising and unfavourable cases; that, where applicable, it is, at least, equally certain of effecting a cure; that where one would be likely to fail from disease of the heart, diseased artery, or other cause of deranged circulation, the other would certainly fail also; nay, there are numerous reasons for believing, that pressure would be safe and successful where an attempt to tie the artery might be regarded as wholly unjustifiable: and all this has been substantiated by an array of cases occurring under different circumstances, and treated by different persons, sufficient to carry conviction to any unprejudiced mind. And here it may be observed, that the fact of a number of cases being treated on the same plan by different persons, distant from each other in time and place, and circumstance, ought to constitute the most unanswerable argument in favour of the treatment adopted. One individual may be so fortunate—or, if the expression is more grateful, so skilful—as to number ten or twelve of such cases in succession, but under the other circumstances there must be something more, something connected with the remedy, rather than with the hand that administered it. On these points I should despair of making any important additions to the arguments, facts and illustrations already so ably brought before the Profession, and, therefore, shall confine myself to some observations connected with the operation this treatment is intended to supersede.

It cannot be denied that the treatment by compression *appears* to be tedious, although I doubt much, that, on an average, it occupies more time than would be necessary for the management of a case by ligature, from a patient's entrance into hospital until his dismissal. It is also true, that it is troublesome to the attendants until the patient comes to understand his case, and assist in the regulation of the screws: and that it is painful is evidenced by the fact, that patients have, after a short trial, refused (as in the case of Dr. Murray) to permit its continuance. I rather think that this latter occurrence is frequently the result of too powerful a compression, but nevertheless it has happened, and in examining the question, I am disposed to look at it in its worst aspect, and accord to every objection its full value. These facts

* See a Paper by Dr. Bellingham, Dublin Medical Press, vol. xiii. p. 181.

being conceded, then, it follows, that no patient should be exposed to such inconvenience, unless for the purpose of escaping something worse,—some suffering either more intolerable in its nature, or more perilous in its results, and therefore, it becomes necessary to prove that the operation of throwing a ligature around a large vessel is not that safe, and simple, and easy proceeding, which many would fain persuade us, but that it is really difficult of performance, and pregnant with danger in its results. But the question of the difficulty of any operation is one that may not admit of a general answer, for there is such variety arising from the size, situation, and condition of the vessel to be tied, the cause of the disease, the constitution of the patient, and the capability of the surgeon, that scarcely any two individuals submit to the operation under precisely similar circumstances, and there is this additional fact, that there are pathological conditions, both of the system and of the arteries at present wholly unknown, which often mar our best conducted operations, and lead to results that could neither have been foreseen or avoided. All that can be asserted, then, is that the operations of securing almost all the great arteries of the body have been performed, and with a wonderful amount of success, and whilst it is conceded that different surgeons have acquitted themselves with a greater or less degree of facility, according to their respective acquirements and dexterity, it must be acknowledged, that patients have been lost by ignorance or *mal-addresse.* So far, then, the word "difficult may be legitimately used in a comparative sense, as applied to this as well as to every other surgical operation, but that will not be sufficient for our present purpose, in furtherance of which it will be necessary to show that some of these operations may be "difficult" to any man, no matter how gifted, how dexterous, or experienced, and that cases may and must occur, both numerous and of an important character, in which a failure is nearly unavoidable.

There is a remarkable condition of an artery which I do not recollect to have seen described, although it must be familiarly known, and doubtless has often been a source of perplexity to every operating surgeon: it is where it forms an abnormal but close adhesion to its adjacent structures, and particularly to its accompanying vein. Every surgeon, in operating, must have observed that it was more difficult to detach from its connexions and to denude an artery in some patients than in others; and the dissection of dead subjects will exhibit the same phenomenon, if carefully sought for; but in the latter case it is generally overlooked, either because attention has not been sufficiently directed to it, or that it may not have been regarded as practically important. I scarcely know whether this can be truly called a pathological condition of the artery, being in reality a product of disease. I am wholly uninformed as to its exciting cause, its progress, and its course; and it would only be a conjecture to attribute it to some species of chronic inflammation rendering the cellular coat of the artery thicker and shorter, and disposing it to contract close adhesions to every surrounding structure. However explained, the result is but too evident in many cases where arteries and veins lie in close juxtaposition, and become so mutually adherent as not to permit of separation even by the knife; or, as I have heard it forcibly expressed by a distinguished anatomist, "to possess but one and the same wall." This condition I was formerly in the habit of regarding as existing only between the popliteal artery and vein, where I had heard it spoken of, and seen it demonstrated by anatomists as the natural and ordinary state of the parts; but some years since I was led to modify that opinion by observing, in the dissection of a case of axillary aneurism, this very adhesion established between the subclavian artery and vein at the only point where the vessels came in contact, and where it must have proved a source of insurmountable embarrassment had an operation been attempted. I have since seen it in the axilla in the person of a cripple who had been obliged to use crutches for many years; its existence in the thigh is by no means unfrequent, and I believe it may occur between any artery and vein in the extremities, although, for reasons with which we are at present unacquainted, it prevails in some localities and some vessels more than others. A curious, and perhaps interesting, speculation might be opened here, as to how far this state of chronic inflammation (if it be really so) might dispose to aneurism, and therefore be most likely to be met with in the very cases where it would prove most embarrassing; or whether it might not explain the frequency of the disease in particular situations, in the popliteal space for instance; or again, whether the altered condition of the cellular coat of the vessel might not render it more brittle, and, by giving it a tendency to break down under a ligature, favour the occurrence of secondary hæmorrhage. Such considerations may possibly form grounds for future pathological inquiry, but, in the present state of our knowledge, could (as I have said) be only speculative, and I willingly forego them for matters more immediately connected with the question at issue.

The femoral artery has probably been the subject of operation as often as all the other arteries of the body taken together; and at the place where it has usually been taken up, it is, in its anatomical relations, rather unfavourably situated, being in front of the vein, which it keeps out of the operator's view. This position of the parts has been constantly brought forward, in explanation of the frequency of the vein being wounded and partially included in the ligature, and the accident, as it was called, uniformly laid to the account of anatomical ignorance, or a want of sufficient surgical dexterity. But this very abnormal adhesion which I have alluded to exists with great frequency between these vessels, and, when it does so, it will furnish a more satisfactory explanation of the occurrence; for, under the circumstances, there is but one wall between them, and in any attempt to pass a needle around the artery, it must be pushed through the vein; the ligature, if tied, must include a portion of it, and occasion an inflammation, that, according to my observation, is certainly and uniformly fatal. Now, let us not, in our own vanity, assert that this is an instance of awkwardness on the part of the operator, and might be easily avoided; neither let us boast our own individual success, which, after all, may be more justly attributable to the accident of not having met with a vessel in this diseased condition. I have seen it happen so frequently, and in such hands, that I feel convinced there must be something more than accident, or ignorance, or carelessness, to occasion it. I know that it has been done by the most dexterous and best-informed men—surgeons constantly in the habit of performing bold and difficult operations, and who never, in other cases, were accused of *mal-adresse.* I once heard a surgeon, whilst in the act of performing the operation, speak of the occasional adhesion of the vessels, and adduce it as a reason why he should proceed with a more than ordinary degree of slowness and precaution, yet he passed his ligature through the vein notwithstanding, and eventually his patient died. I saw another who, in passing his needle round the artery, obviously wounded the vein, for about a spoonful of very dark blood welled up from the bottom of the wound: he was aware of what he had done, withdrew the instrument, passed it higher up, and, in the second attempt, actually included and tied a portion of the vein. Nay, I have been present when a lecturer, engaged in demonstrating the operation on the dead subject, and dwelling on the possibility of committing this unfortunate mistake (as he called it,) practically fell into it himself by opening the vein: yet here were no cries or struggles to embarrass the operator, no impatience of suffering to occasion anxiety or haste, no blood to obscure his view, nothing to prevent him from calmly and quietly separating the vessels, except the one pathological fact, namely, that they were inseparable. But I may not pursue this line of illustration farther. Surgery has not yet arrived at that independence of public opinion that can allow its failings or its faults to be given to the world, and its practitioners are chary of having their unsuccessful cases even distantly alluded to. I shall, therefore, only refer my readers to their own observation and experience, and allow them to say whether such casualties have not been but too frequent, although they may not have been explained according to the views I have ventured to adopt.

But any difficulty that may attach to the performance of this operation weighs as nothing in the scale when compared with the dangers attendant on it,—dangers which occur whether the patient is strong or weak, powerful in constitution or debilitated and broken down, and whether the operation is performed with consummate dexterity or the reverse. I pass the consideration of erysipelas, diffuse inflammation, and that host of similar maladies which so often baffle our best exertions, and confining myself to the accident of secondary hemorrhage alone, which seems to be almost proper and peculiar to this operation, I ask those who confidently vaunt an uninterrupted and uniform success, whether there is nothing to be dreaded from this source? The answer will be found in the multitude of supposed causes to which this unhappy occurrence was attributed, and in the corresponding number of precautions that were suggested, and uselessly suggested, for its avoidance—and still more forcibly in the fact, that the Hunterian operation was practically neither so satisfactory nor so successful, but that all looked on it with some degree of apprehension, and many, in seeking some mode of treatment that might be substituted for it, occasionally and accidentally performed cures, without knowing how or why, or being able to establish a principle that might govern the conduct of others. Thus, many surgeons may possibly be entitled to claim the honour of having anticipated Mr. Hutton in the treatment of aneurism by compression, applied on the trunk of the artery; and even the records of surgery prove that the practice is not altogether new. We read in the *Dictionnaire des Sciences Medicales* of a certain merchant in the Isle of St. Louis, who cured himself of a popliteal aneurism, by condemning himself (such is the expression,) during an entire year, to the most absolute rest, confinement to bed, rigid abstinence, frequent blood-lettings, and compression of the crural artery against the femur, at the situation of its passage through the triceps muscle. This latter was effected by means of a half circle of steel, resembling the spring of a truss, whilst a pad, acted on by a screw, enabled him to graduate and regulate it at pleasure. At first the pain was too severe to permit of the pressure being constantly maintained, but as he became accustomed to it, he was enabled not only to sustain, but to increase it, until the pulsation of the tumour diminished in force, and at length ceased altogether. This case bears a striking resemblance to many of those recently treated, in the instrument employed, and indeed in every particular, except the length of time occupied in the cure, but it does not seem to have attracted any marked attention at the time, or to have been followed by any beneficial result. Again, the idea of curing an aneurism by a gentle and gradual diminution of the force of the circulation in the tumour, has no claim to novelty either. Dubois managed several cases in this manner, his object being to allow time for the collateral circulation to become sufficiently enlarged to ensure the safety of the limb: but he laid bare the vessel, and accomplished his object by the use of a *presse artere*, and, therefore, his operation, either in principle or execution, could only have been useful in shewing that the total and sudden withdrawal of the impulse of the heart from the blood in the aneurismal sac, was not indispensable to its coagulation. Thus, the elements of what may be termed the modern treatment of aneurism were long since occasionally resorted to in practice, but, not being understood, could not be brought into systematic or combined operation. It merely appears that surgeons were fully alive to the dangers attendant on the ordinary treatment of the disease, and that, in their efforts to escape them, they fell upon other resources, suggested by some accidental case. Probably numbers of patients were thus relieved; I know that some in this city were cured, but the event shewed that their attendants either did not reflect on the method by which the disease was removed, or attributed it to some erroneous or insufficient cause.

On considering the various plans and contrivances that were suggested for the purpose of escaping the perils of an operation, and inquiring into the reason why some of them did not succeed, I think one of the principal causes of failure will be found in our familiarity with the ligature, and the confidence we reposed in it: for thus we were led to distrust any proposal unless it accomplished just as much, and precisely in the same manner. Thus, being accustomed to see the tumour collapse, and its pulsation cease instantaneously on the cord being tied, we considered similar phenomena as essential in any other mode of treatment, and if they were not, or could not be procured, we abandoned the entire procedure as incomplete and inefficacious. I have seen this exemplified within my own experience. Several years since, in consequence of some operations performed at the Meath Hospital being followed by secondary hemorrhage, trials were instituted of other modes of treatment, and, amongst the rest, of that by pressure on the trunk of the vessel. But the attempts were made by methods designed and calculated to produce the effects above stated. My ingenious friend, Mr. L'Estrange, who has since done so much for the surgery of lithotrity and hernia, then contrived a kind of tourniquet to compress the femoral artery at the groin without interfering with any other part of the limb, and well can I recollect the sanguine expectations entertained of the efficacy of this instrument, as well as the disappointment experienced at its failure. Yet it was in every respect perfect for the purpose it was intended to answer. When applied, it completely stopped all pulsation in the tumour, and seemed to arrest all circulation through the artery; nay, more, when, for the sake of experiment, it was fitted on the thigh of a dead subject, it resisted the force of a large and powerful syringe, and suffered not a drop of the injection to pass. No doubt was, indeed, with the pathological views then entertained, no doubt could enter any of our minds as to its efficiency, and yet, when brought into practical operation, it was found to be completely useless. As may be imagined, the disress it occasioned was beyond endurance, and it was speedily abandoned, excepting only in the case of one patient, who seemed to possess uncommon fortitude, and in him it excited erysipelatous inflammation in the course of eight-and-forty hours, and was consequently laid aside. Yet was not the fault in the instrument, but in the principle that directed and regulated its application. Capable of completely arresting the circulation, and intercepting the impulse derived from the heart, it could have been made to accomplish these objects partially, or in a minor degree, had such been the desire or the intention with which it was used: but the result proved that no such idea was then entertained, and the instrument was laid aside for ever. Various trials of other apparatus and different modes of treatment were subsequently attempted; but, acting on the same principle, and directed to the same ends, they all failed, simply because they were made to do too much: the ligature, with all its dangers and disadvantages, was again resorted to and maintained its ground; not because of its simplicity or its safety, but because it alone produced effects consonant with the pathological doctrines of the day. I believe this circumstance to have been for a length of time the chief impediment to improvement, and also that it exercises a similar influence even at the present day, inasmuch as I have seen surgeons, without giving themselves the trouble of reflecting on a new proposal, or examining the principle upon which it is based, refuse their confidence to it because it professed to accomplish the desired object in a manner different from that on which they were accustomed to rely.

But the fact is, that the principle on which aneurisms are thus treated was repeatedly developed before our eyes, in the results of many of our operations, although we failed to observe, and, therefore, to render it available. It is essential that every practitioner, still sceptical as to the effects of pressure, and apprehensive that he will be obliged eventually to fall back on the old resources of his art, should rightly understand this position, for some such conviction will be necessary to assure him, not only that pressure on the trunk of the artery has cured the disease in numerous instances, but that such an event ought to be expected in every legitimate case, that is, in every case that would heretofore have been considered as curable by the ligature. It is familiarly known that blood withdrawn from the circulation of a healthy animal possesses, in general, an uncontrollable tendency to become coagulated, and that it will assume that condition, unless interfered with by artificial means; nay, that it requires great and long-continued disturbance, to the extent of breaking down and disorganizing the structure of the fluid, in order totally and effectually to prevent it. Now, the disturbance experienced by the blood contained within an aneurismal sac is occasioned, first, by the action of the heart throwing a wave of blood into it, and then by the reaction of the sac and its coverings returning a part of this back again into the circulation; and we know by experience that this quantum of disturbance is rarely sufficient to prevent coagulation completely, for a portion of the contents of almost every sac is more or less solidified. Again, the reaction of the coverings of the aneurism depends on their elasticity—a dead force, which requires to be acted on before it comes into operation, and, therefore, must bear a relation to the impulse that produces it, so that, if by any means the power with which the blood is thrown into the sac can be reduced, the power by which it is returned must be reduced also, and by so much will the fluid experience less disturbance, and be placed in a condition favourable to coagulation. Lastly, in proportion as the contents of the sac become solid will the impulse it receives, and, of course, its resiliency be weakened (an effect which we see in the feeble and undecided pulsation of old aneurisms:) and thus it happens that, the coagulation of each portion of the blood increasing the facility of the remainder to assume a similar condition, the entire comes to be solidified. In this manner it is not difficult to understand how a sensible diminution of the force of the circulation, if maintained for a given time, can effect the cure of an aneurism, and why it may not be necessary totally to remove it, as used to be the case when the ligature was employed. Unfortunately such a condition is not likely to be obtained without artificial interference; on the contrary, either the original cause that produced the aneurism, or the irritation occasioned by the presence of the disease itself, generally tends to excite the circulation; and if, from any accidental cause, a contrary effect is produced, and its activity diminished, the depression seldom continues a sufficient length of time to accomplish the desired end. Thus, admitting the truth of the hypothesis, that nothing short of the full and perfect action of the heart can maintain an aneurism, and cause its progressive growth, and that it would decrease in size, and ultimately disappear, if that action could be reduced and kept so for a given time, still it should be admitted that circumstances of so favourable a nature seldom arise spontaneously, and, therefore, the infrequency of the natural recovery can be easily explained.

Seeing, then, that Nature is generally inadequate to the performance of these conditions, the next step is to inquire into the means we possess of affording her assistance, and of their probable efficacy. These are two-fold, the medical and the mechanical; and here it may be observed, that we cannot understand the value of Valsalva's treatment, or explain the curative properties of such medicines as digitalis, &c., save on the principle contended for. But then, it may be asked, why recoveries under medical treatment alone are so few and far between, that little or no confidence is practically reposed in it. Almost all patients suffering from aneurism are debarred from exercise, restricted in diet, and removed, if possible, from all opportunities of medical excitement; almost all are subject to medical treatment, and ordered digitalis, or some other medicine, possessed, or supposed to be possessed of the power of reducing the force of the circulation; here the required conditions are at least partially complied with, why do they not recover? The answer to this question must be put into the form of another: are they really so complied with? for, according to my experience, they are not. Few, very few, patients can be induced to submit to them, and perhaps the number is still more limited with whom the purely medical treatment is ever pushed to the extent desired. Within my own observation I never met with one patient who would submit "to preserve his life by rendering life not worth preserving;" not one who would even endeavour to observe the rigidity of abstinence imposed on him, and I have known many to leave the hospital rather than endure a very moderate degree of privation. As to the medical treatment of aneurism by digitalis, and drugs of a similar character, I am not competent to speak so decisively, but I believe few practitioners are disposed to press it energetically, and I have, in numerous instances, witnessed such treacherously destructive effects from it that I should not regret to see it abandoned altogether. Again, it is generally resorted to in the internal forms of the disease, in aneurisms of the aorta, where the vessel is otherwise probably in an unhealthy condition throughout its entire extent, where the rent or aperture in it is very large, and where it is close to, and immediately under the influence of the heart, and it is evident that if recovery in such a case is possible at all, it must be by means and measures more powerful, and longer applied, than might suffice for the cure in a case more favourably situated. Most practitioners entertain strong doubts of the efficacy of any treatment under such circumstances, and will not pertinaciously insist on interfering with their patient's comfort, or opposing his will, without a reasonable expectation of some countervailing advantage. Thus, the medical treatment of aneurism scarcely ever receives a fair trial, and its efficacy is not so much to be estimated by the number of recoveries actually effected as by the quantity of good sometimes obtained, and the length of time to which existence is occasionally prolonged by our present inadequate and imperfect measures: did we possess therapeutic means of decidedly reducing the circulation, or could we safely persevere in their employment, possibly the result might be widely different.

But in cases of external aneurism we have mechanical contrivances to aid us, by which we can graduate and regulate the circulation through the tumour, and reduce it, and keep it reduced, to any point the patient may be able to endure; but as he will not bear its entire interruption, it is necessary to shew that something considerably short of that will be sufficient to cure the disease notwithstanding. Now, the simplest and most satisfactory method of doing this is by proving that it often happened, under the old system of treating aneurism, in cases where nothing of the kind had been intended, and even against the operator's wish, thus:

Almost all the cases in which pulsation returned in the tumour after the operation by ligature, a circumstance of too frequent occurrence not to have been generally observed, and which sometimes created groundless apprehensions in the mind of the surgeon lest his operation should have failed altogether, were literally illustrative of this mode of treatment.

So also were all the cases in which the artery was purposely tied loosely, in the hope of thus avoiding secondary hæmorrhage.

Some of the cases treated by my friend, Mr. Cusack, ostensibly by direct pressure on the tumour, were of this nature also, for, if I recollect aright, at the same time, and in addition to such pressure, he used compression on the trunk of the vessel also.

It is quite evident that in not one of these instances was the impulse derived from the action of the heart wholly removed, in not one was there more effected than a diminution of the force with which the current of blood flowed into the aneurismal sac. This current might have been impelled with an impetus greater or less in different cases, and, therefore, the cure might have been accelerated or delayed, it might have come directly through the imperfectly tied or compressed vessel, or by the collateral circulation; still, in each and in all, it entered the sac with a diminished force and in a diminished stream. In the first case* in which I placed a ligature on the carotid artery the pulsation returned in the tumour in less than four hours, and continued in it for days or even weeks: here the most that was accomplished by the operation was a reduction and, as might be inferred from the progress of the case, a very trifling reduction of the force by which the blood was propelled into it, yet the patient recovered, and, as verified by dissection seven years afterwards, the aneurismal sac was entirely obliterated.† In Sir Philip Crampton's case, treated in the Royal Infirmary, in which

*Dublin Hospital Reports, vol. v.
†Porter on Aneurism, p. 152.

he purposely tied the femoral artery in a very lax noose, he assuredly did no more than diminish the force of the current through it, yet that patient recovered also. Every one of those cases, and many more of a similar character, might be added to the list of those treated by compression on the artery, for the recoveries were based on the same principle, although not effected in the same manner, and any one calmly reflecting on the number and frequency of these, must feel surprised, not alone that the proposal of treating aneurism by pressure should be met with doubt and hesitation, and even rejection, but that a principle obviously, and almost daily, placed under our observation, was not sooner laid hold of and brought into practical operation. True, the disease may occupy situations where this particular mode of treatment cannot be adopted, to which there may be no means of applying pressure, or where there may not be sufficient space for its adaptation, and, therefore, we cannot forego our operations on the carotid, the subclavian, and the iliac arteries; but for the other forms of aneurism, and especially that formidable one, the popliteal, still more formidable because of its extreme frequency, and the fatality that attended its former treatment, we ought to consider ourselves happy in possessing a resource which promises to supersede the necessity of operation altogether. I look forward with hope, and indeed with confidence, to the day when it shall cease to hold a place in operative surgery, when it shall have become (at least in Ireland) a matter of professional history,—the practice of by-gone days,—and when a surgeon shall undertake the management of a case of popliteal aneurism with as much calmness and reliance on the resources of his art, as he lately approached it with apprehension.

Having thus far endeavoured to prove that it is not indispensably necessary that the whole impulse of the heart should be removed from the blood contained within an aneurismal sac, but that a diminution of it, maintained and kept up for a given time, ought to be and will be sufficient for the cure of the disease, it only remains to inquire into the absolute extent to which such diminution should be carried, and the length of time it must be persevered with. Obviously, the answer to the latter part of this question must depend on the dimensions of the tumour, the fluidity of its contents, the disposition of the blood to become coagulated (for I believe individuals differ in this respect,) perhaps on the size of the rent or aperture in the vessel, and a variety of other circumstances and characters in which one specimen of the disease may differ from another; it cannot, then, be determined at once, but it may be observed, that, as far as experience has hitherto shown, the cure is completed in at least as short a time as it ever was by operation, and sometimes even in less. The case* treated by Mr. Cusack seems, as reported, to have

*Dublin Medical Press, vol. xiii.

been as unpromising for any mode of treatment, whether by operation or otherwise, as can be easily imagined, yet it got well in the almost incredibly short space of seven days; but on this point it would be unprofitable to enter on an analysis of the recorded cases, seeing that the actual degree of compression employed in each cannot be ascertained, a circumstance that, along with those already enumerated, must materially influence its necessary duration. But with respect to the other part of the question, it appears to be proved that the requisite diminution of the force of the current of blood is much less than could have been *a priori* imagined, and, consequently, that the pressure on the trunk of the artery may be comparatively gentle. This might have been anticipated from the not unfrequent cures of aneurism by the moderate pressure of tumours in their neighbourhood, and it is a point of the greatest practical importance, because, as has been stated, all our former failures were caused by a degree of compression that could rarely be endured, or, if borne for a time, produced erysipelas or ulceration, or even gangrene, of the parts underneath. Another consideration of some consequence is, that the pressure, however slight at first, may be subsequently increased according as the patient becomes accustomed to it; at least such inference may be deduced from some of the cases recently treated, and it may have some weight with those who still hold to the opinion that the entire removal of the impulse of the heart is, at some period of the treatment, indispensable to the cure. For my own part, I believe, a very moderate degree of pressure, maintained throughout the entire progress of the case, will, in the majority of instances, be found sufficient; that which I employed was barely capable of communicating an impulse from the artery to the instrument, and causing it to vibrate gently in correspondence with the pulsations of the vessel. Under this the limb at first assumed a purplish hue, and, in the course of three or four days, became slightly œdematous, symptoms which, I confess, would have created some alarm, had I not had the advantage of witnessing some of the previously treated cases: it also occasioned pain in different parts of the limb, and this to such an extent in the case of Dr Murray as to induce him to abandon the treatment for a time, though with a firm resolution of recurring to it at some future period. I can easily understand how the occurrence of such symptoms might awaken the most painful anxiety, and even lead to a discontinuance of the attempt. I can comprehend also how the trouble of attending such a case and regulating the clamps and screws might decide a practitioner in declining to undertake it, but it should be recollected that we do not extol the practice as being simple and safe, and free from pain, and exempt from trouble; we only compare it with another, and advocate it on the grounds of being more free from danger, more conservative of human life. I do not, then, pretend to measure the extent to which the compression might gradually be increased, or say how far it was carried by other practitioners, but in the cases I have detailed it never at any time was sufficient to arrest the circulation entirely, or cause the pulsation of the tumour to cease, until it cease altogether and for ever; neither can I conceive how more than a very gentle compression can ever be necessary, if the pathology of the case bears any analogy with, or resemblance to, that in which pulsation returns in the tumour after the artery has been taken up. In this latter case the degree of compression, whatever it may be, is applied at once, and remains afterwards unaltered, and the current of blood, whether it pass through its accustomed channel, or by the circuitous course of the collateral circulation, cannot be mechanically rendered weaker on one day than on the preceding; yet eventually, under the gradual coagulation within the sac, the symptoms disappear and the disease is cured. Thus, we have satisfactory evidence that it never can be necessary to apply such a degree of pressure as would induce any really dangerous condition of the parts, even if it could be quietly endured, and we have also testimony that a very moderate one will be sufficient, in the interesting fact, that in a great number of our cases the regulation and management of the clamps and screws were, after a very short time, intrusted to the patients, who would not be very likely to inflict pain upon themselves, or enforce the remedy beyond that which could be easily endured.

But it has been stated to me in more than one communication on this subject, that the practice had been tried and failed, and that it was necessary, in such instances, to fall back again upon the old operation by ligature. The validity, or rather the truth of this objection, must be conceded at once, inasmuch as I know of a similar occurrence having taken place in Dublin, but when examined it really will not appear to be of much importance. Let us make allowance for the difference of temper observed in individuals—for the irritability and impatience often exhibited under circumstances of far less severity. Again, let us recollect how very recently the systematic treatment of aneurism by compression has been introduced; that our instruments are probably far removed from the degree of perfection that may hereafter be attained; that there are no certain rules or precepts to direct their application; that they may have been used irregularly, imperfectly, or even injuriously; or, supposing them correctly applied, that they may have been hastily and prematurely discarded, and then the only matter for surprise will be that the failures have not been more numerous, and the objections more loudly pressed. But is it exactly fair thus to criticise a proposal confessedly so new? Is it just to expect from a practice barely in its infancy, the full perfection of a ripe maturity? The history of surgery, and particularly of the operative part of it, might teach a different lesson; even the Hunterian operation itself, clumsy and unskilful in the outset, required time and patience, and pathological investigation, to raise it to its present estimation—a position it never would have reached had it been judged by the result of its first and earliest trials. But there is a totally different point of view in which this objection may be legitimately placed, and in which the case of Dr. Murray appears to be particularly valuable. Here is a medical gentlemen, not likely to be mistaken in his own case; he is seen by a number of surgeons perfectly conversant with aneurism, and the nature of his disease is fully recognized; he is subjected to a particular line of treatment for twenty days, becomes impatient under the pain, gets up and resumes his avocations; not only does not pursue any measures that might possibly favour a recovery, but adopts habits of a directly contrary nature, taking long walks, and using a great deal of exercise, and the disease disappears notwithstanding. Now I am not in a condition to prove that the compression cured this patient, but the contradictory of this proposition is equally insusceptible of proof; and if it did cure him, which is at least within the range of possibility, is it not equally possible that some of those patients who had been operated on after compression had apparently failed, might have experienced a similarly fortunate result? I am not disposed to place any very great stress on this solitary case. I know how very little reliance can be attached to insulated facts, and how dangerous it is to reason from them; still, in the investigation of a new proposal, which, if established, must be a decided improvement in practical surgery, it cannot be unfair to bring forward any fact that seems even remotely to bear upon the subject. Let it and all others be subjected to the rigid ordeal of experience, the only test that will ever determine the value of any practical suggestion: if false, their worthlessness will soon be made apparent; but if true, they will establish for themselves a hold on professional opinion not to be assailed by argument or speculation, however plausible or specious.

*Dublin Quarterly Journal of Medical Science.*

